# Exploring the Molecular Pathogenesis, Pathogen Association, and Therapeutic Strategies against HPV Infection

**DOI:** 10.3390/pathogens12010025

**Published:** 2022-12-23

**Authors:** Anirban Goutam Mukherjee, Uddesh Ramesh Wanjari, Abilash Valsala Gopalakrishnan, Sandra Kannampuzha, Reshma Murali, Arunraj Namachivayam, Raja Ganesan, Kaviyarasi Renu, Abhijit Dey, Balachandar Vellingiri, D. S. Prabakaran

**Affiliations:** 1 Department of Biomedical Sciences, School of Biosciences and Technology, Vellore Institute of Technology, Vellore 632014, India; 2 Institute for Liver and Digestive Diseases, College of Medicine, Hallym University, Chuncheon 24252, Republic of Korea; 3 Centre of Molecular Medicine and Diagnostics, Department of Biochemistry, Saveetha Dental College & Hospitals, Saveetha Institute of Medical and Technical Sciences, Saveetha University, Chennai 600077, India; 4 Department of Life Sciences, Presidency University, Kolkata 700073, India; 5 Stem Cell and Regenerative Medicine/Translational Research, Department of Zoology, School of Basic Sciences, Central University of Punjab, Bathinda 151401, India; 6 Department of Radiation Oncology, College of Medicine, Chungbuk National University, Chungdae-ro 1, Seowon-gu, Cheongju 28644, Republic of Korea; 7 Department of Biotechnology, Ayya Nadar Janaki Ammal College (Autonomous), Srivilliputhur Main Road, Sivakasi 626124, India

**Keywords:** HPV, coinfection, microbiota, treatments

## Abstract

The human papillomavirus (HPV), commonly documented as the cause of warts, has gained much interest recently due to its possible links to several types of cancer. HPV infection is discussed in this review from multiple angles, including its virology, epidemiology, etiology, immunology, clinical symptoms, and treatment. Recent breakthroughs in molecular biology have led to the development of new methods for detecting and treating HPV in tissue. There is no cure for HPV, and although vaccines are available to prevent infection with the most common HPV viruses, their utilization is limited. Destruction and excision are the primary treatment modalities. This review sheds light on the epidemiology, molecular pathogenesis, the association of several other pathogens with HPV, the latest treatment strategies available to treat the same, and an overview of the progress made and the obstacles still to be overcome in the fight against HPV infection.

## 1. Introduction

The human papillomavirus (HPV), the most common sexually transmitted infection (STI), significantly negatively impacts a person’s social life. Sexually active people will get an infection at least once without developing any pathologies [1,2]. HPV is a double-stranded DNA virus that causes cervical, anal, vulvar, vaginal, and penile cancers, among several other types [3,4]. Globally, viral infection exhibits a wide range of geographic, socioeconomic, cultural, and genetic variation, as well as inherent individual differences such as age, gender, anatomic site, and health status [5]. These distinctions can be seen in the epidemiologic distribution of HPV infection and its associated disease burden. According to reports from around the world, the three types of HPV vaccines currently available—bivalent, tetravalent, and 9-valent—effectively reduce the incidence of HPV infection and HPV-related diseases [4,6]. Their effectiveness is due to their ability to target and induce immunity against the low-risk (LR) and high-risk (HR) HPVs, which cause 70 and 90% of genital and cutaneous warts and cancers, respectively. Despite the vaccines’ efficacy, the prevalence of HPV-associated pathologies remains high. Epidemiological surveillance of HPV infection, associated conditions, and vaccine acceptance is critical for monitoring and assessing the prophylactic antiviral vaccines currently available—the 2, 4, and 9-valent vaccines [7,8,9].

## 2. Epidemiology of HPV Infection

HPV infection is higher in developing countries. The combined studies of cytologically healthy women found that Sub-Saharan Africa (SSA) (24.0%), specifically Eastern Africa (33.6%) and Latin America (33.6%), had a higher HPV prevalence than the rest of the world [1,7,10,11,12]. The Asian regions had the highest HPV prevalence in females, with nearly half Central and Southern Asians (57.7 and 44.4%, respectively) being carriers. The women in Southern and Eastern Africa (42.2 and 32.3%, respectively) were HPV carriers. HPV prevalence was low (30%) in almost all European countries and low (3.7%) in Western Europe. As a result, developing countries have a higher rate of HPV infection (42.2%) than developed countries (22.6%). Despite this, the prevalence is low in North Africa (9.2%) and Western Asia (2.2%) and moderate in Eastern Europe (21.4%). Furthermore, age-related trends were consistent across all of these studies with female participants [13,14,15].

Men have a global prevalence rate of genital HPV infection ranging from 3.5 to 45%, whereas women have a prevalence rate of 2–44%, with similar transmission rates. Anogenital HPV is primarily transmitted through sexual contact. According to a 2014 study, 9.0% of 4065 healthy men from Europe, America, Asia, and Africa had an HPV infection. Homosexuals and HIV-infected men are at higher risk of HPV anal infection, with a higher incidence rate (90%) than heterosexual men, whose risk of HPV infection is determined by the number of sexual partners [1,16,17]. Furthermore, the rate of HPV infection in all men varies very little with age and is high in both young and older men. In terms of geographic distribution, men are more likely than women to contract HPV in Africa, particularly among South African men (17.2% per year) and Asian men (3.2% per year) [1,13,18,19,20].

Cutaneous warts (CWs) are caused primarily by the genera β and γ (HPV4 and 65), with sporadic contributions from the genera HPV2, 27, and 57, and HPV1 from low-risk oncogenic genital HPVs (LR-HPV), also known as cutaneous HPVs [21,22,23]. A recent study in Dutch children revealed a high cutaneous HPV rate of 92% in CW samples. More than half of healthy people have HPV-containing commensal cutaneous flora [24]. As a result, the persistence of viral multiplication causes CW to manifest, though it only lasts a few months. The prevalence of CW in healthy subjects is less than 33% and is higher in men [25,26].

## 3. The Molecular Pathogenesis of HPV Infection

### 3.1. The HPV Infection Cycle

The viral antigens and host receptors interact at the molecular level to allow the virus to enter the undifferentiated epithelial cells. Structural studies have revealed L1-specific heparan-sulfate proteoglycan (HSPG) binding sites that are high in lysine (K), which is required for productive infection [1,27,28]. Once in the intraepithelial environment, the L1 and the HSPG bind for the first time. It is worth noting that L1 proteins bind to HSPGs via their K278-K361 apolar site, found on the FG and HI surface loops of two nearby pentamers; non-specific binding also occurs with non-HSPG receptors [Laminin 332 (LN332)] [1,26,28,29,30].

Endocytosis allows the virus to enter the cell and travel inside small vesicles to the ER and Golgi. It undergoes a series of interactions and structural changes that will enable the viral genome to be released close to the nuclear membrane. Nuclear pores let the episomal viral genome enter the nucleus and begin viral replication. HPV replication requires epithelial cell differentiation. Indeed, the virus ensures its persistence and multiplication by affecting undifferentiated basal cells. The basal cells consistently provide sequential viral protein synthesis while dedifferentiating. As a result, viral replication (latent or active) will occur, increasing the risk of HPV infection and associated diseases [31,32]. As a result, the L1 protein is essential to the infection process. The abundance of surface epitopes facilitates its potential to boost HPV immunity by producing many highly effective and specific antibodies that recognize HPV in the physiological medium. Given this divergence, developing vaccines specifically from L1 proteins for each region would be an effective initiative to eradicate HPV infection.

### 3.2. Variability in the HPV L1 Protein and the Pathophysiology

Small double-stranded DNA viruses with a diameter of 50–60 nm and no envelope are members of the Papillomaviridae family. The viruses’ 72 capsomeres are copies of pentameric monomers composed of five identical L1 proteins that anchor one L2 protein [13,33,34]. The extended terminal region, a non-coding region, the eight functional early (E1-E8) and two structural late (L1 and L2) proteins are all carried by only one strand of the 7–8 kb circular genomic DNA. The 55 kDa major capsid protein (L1) consists of variable and constant regions encoded by 1.7-kb open reading frame (ORF) [35,36]. The latter is unique to each HPV genotype and consists of surface-specific antigenic epitope-carrying loops that engage with the host’s membrane receptors during cell entry and produce neutralizing antibodies. Despite residual variability, these loops have a consistent three-dimensional structure within HPVs [1,35,37]. In the highly conserved regions of identical HPV types, the latter plays the same roles as the former, including membrane receptor binding and L1-L1 and L1-L2 interactions [1,38].

## 4. Pathogen Association with HPV

### 4.1. Chlamydia Trachomatis Coinfection with HPV

The two most prevalent factors in sexually transmitted diseases worldwide are HPV and *Chlamydia trachomatis*. Additionally, *C. trachomatis* may enhance the risk of HPV infection and support viral persistence [39]. *Chlamydia* obstructs HPV-induced processes, such as mismatch repair (MMR), that maintain cellular and genomic integrity in coinfections. During coinfections, inverse modulation of MMR is distinguished by distinct post-translational proteasomal degradation and E2F-mediated transcriptional regulation [40].

Interestingly, despite causing DNA damage by producing reactive oxygen species [41], *Chlamydia* superinfection of these cells reduces the E2F-mediated control of DNA repair pathways and quiescence [42]. HPV E6E7 and *C. trachomatis* induces different host cell transcriptional pathways, according to a global transcriptome study. The development of *C. trachomatis* is slowed by HPV E6E7 by preventing the RBs from re-differentiating into EBs and inducing persistence. Both pathogens upregulate the immune response mediated by tumor necrosis factor (TNF), while *C. trachomatis* explicitly induces the inflammatory response mediated by IL-17 and NF-kB signaling. Additionally, oxidative phosphorylation, RNA regulation, RNA processing, and elevated MAPK pathways are all significantly affected by chlamydia [40].

### 4.2. Treponema Denticola Chymotrypsin-like Protease (Td-CTLP)

Td has several virulence factors, such as attaching to epithelial cells and extracellular matrix elements, releasing degrading enzymes, producing cytotoxic chemicals, activating host-derived tissue-destructive matrix metalloproteinases, and inhibiting local immunological responses [43,44]. CTLP is one of its primary virulence factors, breaking down many basement membrane components and allowing Td to infiltrate epithelial tissue [45]. A study by Kylmä et al. reported that Td-CTLP detection in 81% of the oropharyngeal squamous cell carcinoma (OPSCC); precisely, 48% of all HPV-negative and 52% of HPV-positive OPSCCs. The high TLR 5 and TLR 7 are linked with Td-CTLP expression in HP-negative OPSCCs. Their investigation stated that there is no information on whether HPV infection is followed by Td-infection or vice versa in HPV-positive or Td-CTLP-positive cancers. Furthermore, a detailed study is required to understand the role of Td in HPV-positive OPSCCs [44].

### 4.3. Coinfection of HPV and HIV

At the molecular level, HPV and HIV interact, and one can lead to an infection with the other. Both men and women are more likely to contract HIV if they have an HPV infection, and HIV-infected people have a higher risk of developing cancer and dysplasia from HPV due to immune system suppression [46]. The human papillomavirus (HPV) has been identified as a prominent co-infecting virus with HIV infections in many areas of Africa and other continents where it is endemic. According to extensive observational studies involving HIV-positive women, cervical intraepithelial neoplasia (CIN) correlates strongly and consistently with HIV and HPV coinfection [47,48]. According to estimates, HPV infection affects three out of every four HIV-positive women. An HIV viral load and CD4 levels correlate with HPV infection rates in HIV-positive females [49,50]. At the molecular and cellular levels, HIV infection favors HPV during the many stages of the HPV cycle, including HPV immunological escape from host defenses, HPV penetration into the target cell, and HPV replication [46].

In a study conducted in Romania’s southeast region, it was observed that the HPV infection is widespread among HIV-positive women and is correlated with factors like age at first sexual experience, several partners, CD4 count, vaginal candidiasis, as well as the Gardnerella infection [51]. It is becoming more and more clear how HIV and HPV interact, its molecular mechanism, and how the immune system interacts with both these viruses. It was noted that rate of HPV clearance is slowed down, and the oncogenic risk is increased by the immunological failure brought on by HIV infection [52]. Another study showed that the most common lesions among women with HIV and HPV co-infection, high-grade squamous intraepithelial lesions (HSIL), had a prevalence of 27.38% [53].

### 4.4. Coinfection with Other Pathogens

The pathogen that causes gonorrhea, *Neisseria gonorrhoeae*, is an obligate human pathogen [54]. *T. vaginalis* infection is a frequent and possibly dangerous infection. Infection with this protozoa is increasingly understood to be linked to reproductive tract problems, such as post-abortal infection, post-cesarean infection, preterm birth, and premature rupture of membranes, in addition to reproductive tract discharge and discomfort [55]. *Trichomonas vaginalis* and HPV infections are not notifiable. *N. gonorrhoeae*, *C. trachomatis*, *T. vaginalis*, and HPV were frequently discovered in 53% of female patients at an urban STD clinic in Mongolia, according to a study [56]. HPV was frequently detected (36%) along with the traditional sexually transmitted infections (STIs) *N. gonorrhoeae*, *C. trachomatis*, and *T. vaginalis*, with oncogenic genotypes being present in 44% of positive cases [57]. One study found that males in central Australia had a 20.9% prevalence of *C trachomatis* and *N gonorrhoeae* [58]. Without considering HPV, 37% of the people in the study had *gonorrhea*, *chlamydia*, or *trichomonas* infections. It is a high percentage, especially considering that traditional STIs increase the risk of HIV transmission [56].

## 5. The Role of microRNAs in HPV

MicroRNAs (miRNAs), one of the many classes of biomolecules dysregulated by HPV, have recently emerged as essential carcinogenesis regulators that can regulate intricate processes like cancer metastasis. Multiple cellular miRNAs may have a part in the development of HPV-related cancer, according to in vivo research using mouse strains that are HPV-16 transgenic [59]. High-risk HPV is identified in 99.7% of cervical malignancies, and there is a strong correlation between ongoing infection with high-risk HPV and cervical cancer (CC) [60]. Lymph node metastases and an advanced clinical stage of CC are also linked to elevated levels of circulating miR-21 in patients [61]. In order to find miRNAs that can predict the existence of CIN3 and CC in self-samples, a study was carried out on the genome-wide miRNA profiles in HPV-positive self-samples. This investigation showed that deregulated miRNA expression was linked to the emergence of CIN3 and CC [62,63,64].

## 6. The Role of Exosomes in HPV

Compared to exosomes from HPV-negative cells, those from HPV-positive cells have better angiogenic potential and a higher rate of cellular uptake by endothelial cells. Increased expressions of PTCH1, VEGF-A, VEGFR2, and angiopoietin-2 are found when the underlying processes causing the angiogenic potential of HPV-positive exosomes are examined [65]. In 2009, the discovery of extracellular survivin in HPV-18-positive HeLa cells provided the first evidence of the role of extracellular vesicles (EVs) in HPV pathogenesis [66]. Exosomes from HeLa cells with the E6 and E7 proteins silenced had fewer inhibitor of apoptosis protein (IAPs), even though they secreted more exosomes overall than control cells. This demonstrated that the presence of IAPs was dependent on HPV oncoproteins [67]. mRNA, miRNA, and cytokines could all be transferred horizontally between cells using exosomes produced by HPV-infected cells. As a result, exosomes possibly influence the immune system in the CC microenvironment. Through the Wnt signaling pathway, the proinflammatory cytokine IL-36γ can cause inflammation in keratinocytes [68,69,70,71].

## 7. Deintensification for HPV Treatment

De-intensification, or lowering the effects of the cancer therapy regimen in some way, is a hot topic in HPV-positive cancer research, although few de-intensification trials have been conducted in recent years. A study in 2017 conducted a phase II trial where they evaluated the complete clinical response towards induction chemotherapy to investigate whether they could select patients with HPV-associated oropharyngeal squamous cell carcinoma (OPSCC) for a reduced radiation dose, which might help in impeding the disease progression [72]. This clinical trial administered cetuximab after reduced-dose radiation, improving the treatment results. Another clinical phase II trial in HPV-associated OPSCC studied the survival rate of progression-free patients using the de-intensification strategy, which supported progression onto the phase III trials [73]. This study’s de-intensification of the radiation dose provided positive results, suggesting its significance in HPV-associated cancer treatments. Other clinical trials were also conducted in OPSCC [74,75,76]. Due to physiologically and demographically unique conditions, patients with HPV-associated OPSCC have significant cure rates following conventional therapies. De-intensification techniques are an essential research topic due to conventional therapies’ long survivability time and toxicity; nevertheless, they should be tailored to specific factors related to the disease biology for each patient [77].

## 8. HPV Preventive Strategies: Challenges

The incidence of HPV infections and the associated diseases keeps increasing. Advanced prevention strategies are required to eradicate viral infections. HPV is commonly transmitted sexually. About 300 million women are carriers of the HPV virus [78]. Most HPV infections are undetectable; chronic HPV infection has been linked to tumors and genital warts. Preventive care includes health education and HPV vaccination, whereas secondary prevention concentrates on early diagnosis. Tertiary prevention includes diagnosis, treatment, and palliative care [79].

Although there is no cure for HPV, cytologic testing has been essential in treating CC since the 1950s [80]. To manage HPV infections, the WHO CC elimination strategy has suggested three complementing elements to be realized by 2030. These three pillars include the vaccination of 90% of girls by age 15, the high-performance cervical screening of about 70% of women between the ages of 35 and 45, and the treatment of 90% of cervical disease cases identified [81]. HPV vaccinations are safe and effective in lowering high-grade lesions and CC in approximately 97% of young women vaccinated at 12 to 13 years [82]. Randomized trials consistently show that HPV primary screening provides more robust protection from developing precancers and malignancies than cytology-based screening. Indeed, Pap cytology is progressively supplanted as the primary screening method for women with better sensitivity.

### 8.1. Vaccine Evaluation for HPV Infection

Vaccines were developed to protect against HPV infection and the subsequent onset of HPV-associated disorders. Although three different vaccinations have been clinically produced, not all of them are readily available everywhere. These vaccines range in the number of HPV strains they can contain and target [83]. The quadrivalent vaccine targets HPV types 6,11,16, and 18, while the HPV 9-valent vaccine targets the same HPV types targeted by the quadrivalent vaccine and the HPV types 31, 33, 45, 52, and 58. The bivalent vaccine targets HPV types 16 and 18 [84]. Generally, it is assumed that boys and men should receive vaccinations. Males serve as infection vectors even though the long-term effects of HPV infection in men are often less severe. Herd immunity would be increased, and the CC incidence would be reduced overall if men and boys were included in vaccination programs [85]. HPV vaccinations significantly decreased the occurrence of HPV-related fatalities among girls, women, and boys, according to a recent meta-analysis that included 60 million people from 14 different high-income countries [86]. Studies also revealed that the effectiveness of the HPV vaccination on HPV-associated cancers varies depending on geographical locations [87]. One of the most significant risk factors for HPV infection is the number of sexual partners. The HPV vaccine did not result in a statistically significant increase in sexual activities between men and women who received it and those who did not [84,88].

### 8.2. Live Vector-Based HPV Vaccines

Prophylactic VLP (virus-like particles)-based vaccines have some disadvantages. These include the high cost and the presence of a suitable cold chain. These limitations have prevented its widespread usage in underdeveloped or resource-poor nations, where there were 80% of CC cases [89]. As a consequence, various studies have focused on determining live vector-based vaccines. Live vaccines might be an effective method for avoiding viral infections. Intracellular bacteria like Rubella or Vaccinia viruses and *Listeria*, *Shigella, Salmonella,* and *Lactococci* have been extensively used to make the HPV vaccine [90]. The development of the HPV vaccination using bacterial and viral vectors has shown to be a significant issue for human safety. It was demonstrated that live *Leishmania tarentolae* would be an effective vector for the delivery of the HPV16 L1 antigen and the induction of strong humoral immune responses, thus presenting a powerful immunization approach against HPV and other intracellular diseases [91].

#### 8.2.1. Bacterial Vectors

Due to their convenience and high immunogenicity, live bacterial vectors are used for therapeutic HPV vaccines and are one of the high-potential vector-based vaccines. *Salmonella, Lactobacillus lactis, and Listeria monocytogenes (L. monocytogenes, LM*) are today’s most prevalent bacteria-based tumor immunotherapy vectors [92]. In previous investigations, a plasmid-based complementation approach was used to deliver the HPV E7 gene, but there was a high risk of plasmid loss and the persistence of antibiotic resistance genes in human clinical trials [93]. Recent research suggests that administering vaccination antigens via live-attenuated bacterial pathogens leads to problems, particularly for children, the elderly, and immunocompromised people [94]. When comparing vaccinations using lactic acid bacteria to *Salmonella* and Listeria against HPV, data have demonstrated a wide range of adverse effects for the latter, making lactic acid bacteria vaccinations an effective and promising alternative to the more common attenuated pathogenic bacterial vaccine [95].

#### 8.2.2. Viral Vectors

In order to deliver therapeutic genes to cells for human gene therapy, viral vectors have emerged as crucial tools [92]. Adenoviruses (Ad), adeno-associated viruses (AAV), alphaviruses, and vaccinia viruses are considered the most frequently studied of all viral vectors [96]. A unique and very efficient approach to treating and eradicating HPV-induced malignancies could be viral vectors created to deliver E6- and E7-specific CRISPR/Cas to tumor cells [95]. Studies have shown that high-capacity Adenoviral vectors may be used as HPV-specific cancer gene therapy agents when combined with CRISPR/Cas9, which is HPV-type specific [97].

### 8.3. Peptide and Protein-Based Vaccines

Peptide-based vaccines, one of the most influential vaccine platforms, have received significant research attention as no vaccines have been efficient enough to eradicate pre-existing HPV infections. With the help of non-auto immunogenic antigen sequences derived from cancer-associated proteins, peptide-based vaccines can stimulate cellular immune responses and eradicate CC [98]. Peptide-based vaccinations are MHC-specific; therefore, it is necessary to identify each person’s unique immunogenic epitopes of HPV antigens for the vaccine to succeed. Peptide-based vaccinations must be MHC-specific, which presents difficulties for mass manufacture and managing diseases linked to HPV [92,99]. The fact that protein-based vaccinations include all HLA epitopes is an advantage of their use. A disadvantage of employing peptide-based vaccines is the MHC restriction issue. A therapeutic HPV vaccination called TA-CIN contains the HPV16 E6E7L2 fusion protein [100]. Another therapeutic HPV protein-based vaccination, GTL001 (ProCervix), fights against HPV types 16 and 18 [101].

### 8.4. Antiretroviral Therapy and HPV

Human immunodeficiency virus (HIV) patients are treated with anti-HIV medications as part of antiretroviral therapy (ART). The recommended course of treatment includes a cocktail of medications that prevent HIV replication, frequently referred to as “highly aggressive antiretroviral therapy” or HAART [102]. HAART can potentially change the course of HPV infection through immunological reconstitution of the host. However, information on how HAART affects HPV infection is minimal, and conflicting outcomes have been reported [103].

### 8.5. Listeria Monocytogenes-(LM) Based Immunotherapy

The Gram-positive bacteria *Listeria monocytogenes* (Lm) is most well-known for its capacity to infect people and cause a range of symptoms, which includes gastroenteritis, meningitis, and encephalitis [104]. Listeria monocytogenes has advanced as a vaccine platform for tumor immunotherapy in clinical trials as a result of research on the factors that create Listeria monocytogenes immunogenicity and how these factors might be leveraged. In previous studies, LM-based therapeutic vaccinations that targeted the HPV-16 E7 antigen were created [105]. Increasing low avidity CD8+ T cells with the specificity for E7 that are not destroyed during thymopoiesis can eliminate solid tumors. Listeria-based vaccinations against E7 seem to overcome central tolerance [93].

A virulence component in the Lm life cycle, called listeriolysin O (LLO), pierces the phagosome to facilitate Lm entrance into the cytosol. A study of efficient antitumor immunity employing tLLO-TAA (Truncated LLO- tumor-associated antigens) structures reported that mice injected with HPV LLO-E7-expressing Lm had a complete regression of almost 75% of cancer [106].

## 9. Conclusions

In terms of sexually transmitted diseases, HPV ranks the highest. CC is the most devastating health effect of an HPV infection. In contrast, malignancies caused by human papillomavirus (HPV) also account for a considerable portion of male mortality and morbidity. Further research is needed to understand the course of oral HPV infection more fully. Regular screening for HPVis neither practical nor feasible. Efficacy against prolonged cervical infections, and in the case of the qHPV vaccine, efficacy against anal infections, has been demonstrated for all three preventative HPV vaccines. Campaigns to prevent the spread of human papillomavirus (HPV) should prioritize vaccinating against high-risk and low-risk strains of HPV and reducing the prevalence of HPV disease in people who have previously been exposed to the virus. In recent decades, much progress has been made in understanding how HPVs engage with host cells, tissues, and the immune system. In order to prevent HPV infections, studies have verified and deployed safe and effective prophylactic immunization techniques. An increasing number of people know about HPV and the myriad problems it can cause in women, men, and children; research has also improved the sensitivity and specificity of molecular diagnostic methods for HPV identification that can be used in CC screening. While these successes demonstrate the power of biomedical research to generate vital public health interventions, they also create new and daunting challenges, such as the high price tag associated with HPV prevention and treatment, the difficulty of putting into practice what is technically feasible, the social and political opposition to preventative measures, and the wide variation in economic and health care infrastructure between countries.

## Data Availability

Data are available from the authors on request (A.V.G.).
